# Neurodiversity and mental health in esports

**DOI:** 10.3389/fpsyg.2026.1843950

**Published:** 2026-06-01

**Authors:** Lucas Van Ruysevelt, Olivier Mairesse, Martijn Van Heel, Michael Geoffrey Trotter, Matthew Watson, Ismael Pedraza-Ramirez, Iván Bonilla, Laura Swettenham, Jolan Kegelaers

**Affiliations:** 1 Faculty of Psychology and Educational Sciences, Vrije Universiteit Brussel, Brussels, Belgium; 2 School of Health and Welfare, Hogskolan i Halmstad, Halmstad, Sweden; 3 Institute of Psychology, Deutsche Sporthochschule Koln, Cologne, Germany; 4 Belgian Esports Federation, Brussels, Belgium; 5 G2 Esports, Berlin, Germany; 6 Department of Basic, Developmental, and Educational Psychology, Universitat Autonoma de Barcelona, Barcelona, Spain; 7 National Institute of Physical Education of Catalonia, Universitat de Lleida, Lleida, Spain; 8 Sport and Exercise Sciences, Liverpool John Moores University, Liverpool, United Kingdom

**Keywords:** ADHD (attention deficit and hyperactivity disorder), anxiety, autism (ASC), depression, neurodivergence, sleep disturbance, well-being

## Abstract

**Introduction:**

Both neurodiversity and mental health have become increasingly prominent topics in esports. Nevertheless, little is known about the mental health of neurodivergent esports players specifically.

**Methods:**

The current study, therefore, examined whether neurotype-related differences in mental health outcomes (well-being, depression, anxiety, and sleep disturbance) differed across self-reported competitive tiers through a secondary analysis of a large-scale, international, cross-sectional survey.

**Results:**

A total of 1,075 esports players across competitive tiers and game titles completed the survey, of whom 402 (37.39%) identified as neurodivergent. The analyses showed a main effect of neurotype for well-being and sleep disturbance, a main effect of competitive tier for all mental health outcomes, and an interaction effect for depression and anxiety. Simple comparisons showed that low-tier neurodivergent players exhibited the least favourable mental health profile, reporting lower well-being, higher depressive and anxiety symptoms, and greater sleep disturbance relative to their neurotypical counterparts. In contrast, high-tier neurodivergent players reported comparatively fewer depressive and anxiety symptoms than their high-tier neurotypical counterparts, despite continuing to report lower well-being and greater sleep disturbance.

**Discussion:**

Neurodivergence seems to be a salient dimension of esports participation. A substantial proportion of players in the current sample self-identified as neurodivergent, and competitive-tier based analyses indicated that the mental health profile associated with neurodivergence was not uniform across the competitive pathway. Future research should include longitudinal designs to clarify relationships between neurotype, mental health, and progression/drop-out across competitive levels, adopt more fine-grained approaches in describing and identifying neurodivergence, and capture lived experiences of neurodivergent players.

## 1 Introduction

Neurodiversity has become an increasingly prominent topic within esports (Harrison et al., 2024; Hassan et al., 2024). Neurodiversity refers to naturally occurring variation in cognitive, behavioural, social, and emotional functioning. Relatedly, neurodivergence describes neurodevelopmental profiles which diverge from dominant norms and is “used as a broad term incorporating autism, attention-deficit hyperactivity disorder (ADHD), and other neurodevelopmental conditions” (Hoare et al., 2023, p. 2). Conceptually, the neurodiversity perspective reflects a shift away from deficit-oriented (i.e., medical) views toward a more value-neutral perspective that emphasises natural variation in human neurodevelopment (Chapman, 2021). In other words, variations in neurodevelopmental functioning are ubiquitous and neither necessarily good nor bad (Dwyer, 2022).

Neurodivergence is believed to be very common in esports. For example, in a large population survey, Kegelaers et al. (2025) found that 37.39% of esports players identified as neurodivergent. For comparison, general population estimates suggest that approximately 0.7–3% of children and adolescents receive a diagnosis of autism and around 5–11% receive a diagnosis of ADHD (Francés et al., 2022). One potential explanation for the salience of neurodivergence in esports may be that it aligns with the strengths and preferences commonly reported by neurodivergent individuals. For example, competitive games reward sustained attentional engagement, while the structured, rule-bound nature of games may appeal to individuals who prefer clear systems (Hoare et al., 2023; Kooij et al., 2019). Moreover, ADHD-related heightened attentional engagement and task-focused persistence, including periods of hyperfocus on highly engaging, immediately rewarding activities (e.g., video games), may offer a competitive advantage for players (Kooij et al., 2019). Finally, the online format of many esports may also offer a social environment that suits a wider range of communication styles and sensory preferences, broadening access and participation (Harrison et al., 2024).

Although neurodivergence is in essence a value-neutral construct (Chapman, 2021), this should not ignore the very real distress that can accompany it, much of which may arise from person-environment mismatches rather than from neurodivergence itself (Shah and Holmes, 2023). A substantial body of evidence from the general population consistently shows that neurodivergent individuals are at an increased risk of mental health problems, including depression, anxiety, and suicidality (Hollocks et al., 2019; Meinzer et al., 2016; Polidori et al., 2024). Importantly, social models of neurodiversity suggest that such mental health problems are not due to inherent personal deficits, but rather to interpersonal and environmental mismatches created by a society designed around neurotypical norms (Shah and Holmes, 2023) and may be further exacerbated by stigma and a lack of appropriate support (e.g., Bayeh and Ryder, 2025). Esports players, too, appear to face a substantial mental health burden. Several studies have reported relatively high symptom prevalence rates of common mental health disorders (Birch et al., 2024; Monteiro Pereira et al., 2021). In the most comprehensive study to date, Kegelaers et al. (2025) found that, among 1,105 players, 36.7% reported low well-being, 44.7 and 33.4% reported clinically relevant levels of depression and anxiety symptoms respectively, and 69.3% reported clinically relevant levels of sleep disturbance (Kegelaers et al., 2025). Despite these converging fields of evidence, little is known about the mental health of neurodivergent esports players specifically.

The present study aimed to examine how neurotype and self-reported competitive tier are jointly associated with mental health outcomes in esports. Specifically, we tested whether differences between neurodivergent and neurotypical players in well-being, depressive symptoms, anxiety symptoms, and sleep disturbance varied between lower and higher competitive tiers. By integrating neurotype and competitive tier in the same analytic framework, the study moves beyond simple group comparison and examines whether the mental health profile of neurodivergent players differs across the competitive pathway.

## 2 Materials and methods

To investigate the associations between neurotype, competitive level, and mental health in esports, we conducted a secondary analysis of a large, international, cross-sectional online survey as part of the ‘Good Game’ project. For a description of the recruitment process, see Kegelaers et al. (2025). Ethical approval was obtained from the Ethical Committee of Human Sciences of the Vrije Universiteit Brussel, and data was collected between October 2024 and February 2025.

### 2.1 Material

At the beginning of the survey, participants reported demographic information (gender, age, country of residence), esports background (years of competitive experience, primary esports title, weekly hours of esports engagement), and self-rated competitive level. As objective rankings and competitive structures are not directly comparable across game titles (Poulus et al., 2024), the present study used self-reported competitive level rather than an objective performance metric. These categories were defined through iterative discussions with the project consortium, consisting of both academic partners and applied esports stakeholders. Participants classified themselves into one of five categories; *recreational* (occasionally competing online or at the lowest-tier local events), *foundational* (regularly competing in lower-tier online competitions, local LAN events, events at the bottom of the regional ladder), *pre-elite* (competing in second-tier or regional in-person competitions or online competitions, competing for top-level in-game rankings), *elite* (competing in prestigious tournaments with a good number of top-tier teams, slightly below the highest-level international competitions), or *mastery* (competing in internationally top ranked tournaments, organised by well-established franchises) (for a detailed description, see Kegelaers et al., 2025). Additionally, primary esports titles were descriptively recoded into broader genre categories based on their competitive format. Categories included shooter titles (CS: GO, Rainbow Six, Overwatch, Valorant, Fortnite, Call of Duty), multiplayer online battle arena titles (MOBA; League of Legends, DOTA2), and sports titles (Rocket League, EA Sports FC, NBA2K, and sim racing). As game genre was not part of the primary research question, and some categories contained relatively small cell sizes, genre- and title-based analyses were treated as descriptive.

Self-identified neurotype was assessed using a single self-report item: “Would you describe yourself as neurodivergent? (e.g., ADHD, autism, dyslexia)” (i.e., a formal diagnosis was not needed). Response options were “yes,” “no,” and “prefer not to say.” If respondents answered yes, an optional open-ended follow-up question allowed participants to specify the neurotype they identified with. Open-ended neurotype responses were coded into ADHD, autism, dyslexia and other neurotypes. As participants could report more than one neurotype, responses were also coded descriptively into single neuro-type and multiple neurotype profiles. These co-occurrence categories were used only to describe the sample and were not entered into inferential analyses due to small and uneven cell sizes.

#### 2.1.1 Well-being

Overall well-being was assessed using the World Health Organisation-Five Well-being Index (WHO-5; World Health Organization, 2024). This five-item measure assesses well-being over the previous 2 weeks on a 6-point Likert scale (0 “at no time” to 5 “all of the time”). Item scores are summed (0–25) and multiplied by four to yield a 0–100 total score, with higher scores indicating better well-being. Consistent with prior work, a cut-off of 50 was adopted to assess poor well-being (Topp et al., 2015). Internal consistency of the WHO-5 in the current sample was good (a = 0.83).

#### 2.1.2 Depression

Depressive symptoms were assessed with the Patient Health Questionnaire-9 (PHQ-9; Spitzer et al., 1999), a nine-item measure of symptoms over the past 2 weeks rated from 0 (“not at all”) to three (“nearly every day”). Total scores range from 0 to 27, with higher scores indicating more severe symptoms. A standard cut-off score of ≥10 was used to indicate clinically relevant moderate-to-severe symptoms of depression (Kroenke et al., 2010). Internal consistency of the PHQ-9 in the current sample was good (a = 0.88).

#### 2.1.3 Anxiety

Anxiety symptoms were assessed using the Generalised Anxiety Disorder-7 scale (GAD-7; Spitzer et al., 2006), a seven-item measure of symptoms over the past 2 weeks rated from 0 (“not at all”) to three (“nearly every day”). Total scores range from 0 to 21, with higher scores indicating greater anxiety severity. A standard cut-off score of ≥10 was used to indicate clinically relevant moderate-to-severe symptoms of anxiety (Kroenke et al., 2010). Internal consistency of the GAD-7 in the current sample was very good (*α* = 0.90).

#### 2.1.4 Sleep disturbances

Sleep disturbances were assessed using the Pittsburgh Sleep Quality Index (PSQI; Buysse et al., 1989), a 19-item measure of sleep quality over the past month yielding a global score from 0 to 21, with higher scores indicating poorer sleep. A cut-off score of ≥5 was used to indicate clinically relevant sleep disturbance (Buysse et al., 1989). Internal consistency of the PSQI in the current sample was good (*α* = 0.81).

### 2.2 Participants

In total, 1,105 participants completed the survey as part of the GG project (Kegelaers et al., 2025). Thirty participants chose not to disclose whether they identified as neurodivergent. As such, they were treated as missing, resulting in a final sample of 1,075 participants. Table 1 outlines the descriptive characteristics of the final sample.

**Table 1:** Descriptive characteristics of age, gender, competitive level.

Variable	Level	Total (*n* = 1,075)	Neurodivergent (*n* = 402; 37.39%)	Neurotypical (*n* = 673; 62.61%)
Age		27.86 (SD = 7.61)	26.22 (8.23)	28.83 (7.05)
Gender	Men	900 (83.72%)	322 (80.1%)	578 (85.9%)
	Women	137 (12.74%)	49 (12.2%)	88 (13.1%)
	Non-binary	29 (2.70%)	26 (6.5%)	3 (0.4%)
	Prefer not to share	9 (0.84%)	5 (1.2%)	4 (0.6%)
Competitive level	Recreational	336 (31.26%)	141 (35.1%)	195 (29%)
	Foundational	293 (27.26%)	117 (29.1%)	176 (26.2%)
	Pre-elite	230 (21.39%)	95 (23.6%)	135 (20.1%)
	Elite	147 (13.67%)	35 (8.7%)	112 (16.6%)
	Mastery	69 (6.42%)	14 (3.5%)	55 (8.2%)
Neurotype	ADHD	203 (18.9%)	203 (50.5%)	-
	Autism	105 (9.8%)	105 (25.1%)	-
	Dyslexia	48 (4.5%)	48 (11.9%)	-
	Other	38 (3.5%)	38 (9.5%%)	-

Due to the open-ended nature of the question, participants were able to report multiple neurotypes (e.g., both ADHD and autism) or none at all. In the case of multiple answers, all neurotypes were counted. As such, the percentage of reported neurotype does not equal 100% in the neurodivergent group.

The distribution of primary esports titles and broader game genres as well as their corresponding mean mental health outcome scores, low tier-high tier distribution, and neurotype distribution are reported in Supplementary Table S1. The largest genre category was MOBA (*n* = 412, 38.33%), followed by shooter (*n* = 312, 29.02%), and sports (*n* = 96; 8.93). Additionally, 255 (23.7%) participants reported playing other game titles. These game titles and genre categories were not the primary focus of the study and are reported as descriptive.

### 2.3 Data analysis

All analyses were conducted in IBM SPSS Statistics (version 31). Descriptive statistics were calculated for all variables. Internal consistency was estimated using Cronbach’s alpha (*α* ≥ 0.70 considered acceptable; Nunnally and Bernstein, 1994). Prevalence estimates for poor well-being, clinically relevant depressive and anxiety symptoms, and sleep disturbance were derived using established cut-offs, with 95% confidence intervals (CIs). In line with recommendations to avoid unnecessary dichotomisation (Poucher et al., 2021), continuous scores were used for inferential analyses.

Based on prior work indicating meaningful differences in outcomes between low- and high-level players (Kegelaers et al., 2025), we planned to contrast neurodivergent representation and mental health outcomes based on lower versus higher competitive levels. To investigate neurodivergent representation, we conducted a Pearson’s chi-square, as well as follow-up pairwise 2 × 2 comparisons, between competitive levels. The results of these analyses suggested significant differences in representation across the five levels [*χ*^2^ (4) = 25.52, *p* < 0.001], and similar representation of neurodivergence across recreational, foundational, and pre-elite levels (around 40%) as well as across elite and mastery levels (around 20%) (see Supplementary Table S2). Based on these considerations, and to improve analytic interpretability, we collapsed recreational, foundational and pre-elite level players into a “low tier” group, and elite and mastery level players into a “high tier” group for further analyses.

To examine whether neurotype-related mental health differences varied across competitive tiers, we conducted 2 × 2 factorial ANOVAs for each outcome (WHO-5, PHQ-9, GAD-7, and PSQI). Neurotype (neurotypical–neurodivergent) and competitive tier (“low”–“high”) were entered as between-subjects factors. For each model, both neurotype and competitive tier main effects, and the neurotype × competitive tier interaction effect were evaluated as the primary test of moderation. In addition, estimated marginal means were used to probe group differences through simple comparisons (neurodivergent–neurotypical within both tiers, and “high”–“low” tier within the neurodivergent group), with Holm-Bonferroni adjustment for multiple comparisons. Homogeneity was assessed using Levene’s test, effect sizes for ANOVA effects were reported as partial eta squared (partial *η*^2^), and pairwise contrasts were summarised with mean differences and 95% CIs.

## 3 Results

### 3.1 Descriptive neurotype profiles

Among neurodivergent participants, 218 reported a single neurotype and 83 reported multiple/co-occurring neurotypes. The most common single-neurotype profiles was ADHD (*n* = 126), followed by autism (*n* = 49), and dyslexia or learning-related profiles (*n* = 27). The most common co-occurring profiles were ADHD + autism (*n* = 44), followed by ADHD + learning-related conditions (*n* = 14). Descriptive profile prevalences and their corresponding mental health outcomes are shown in Supplementary Table S3.

### 3.2 Neurodiversity and mental health outcomes: group comparisons

**Figure 1:**
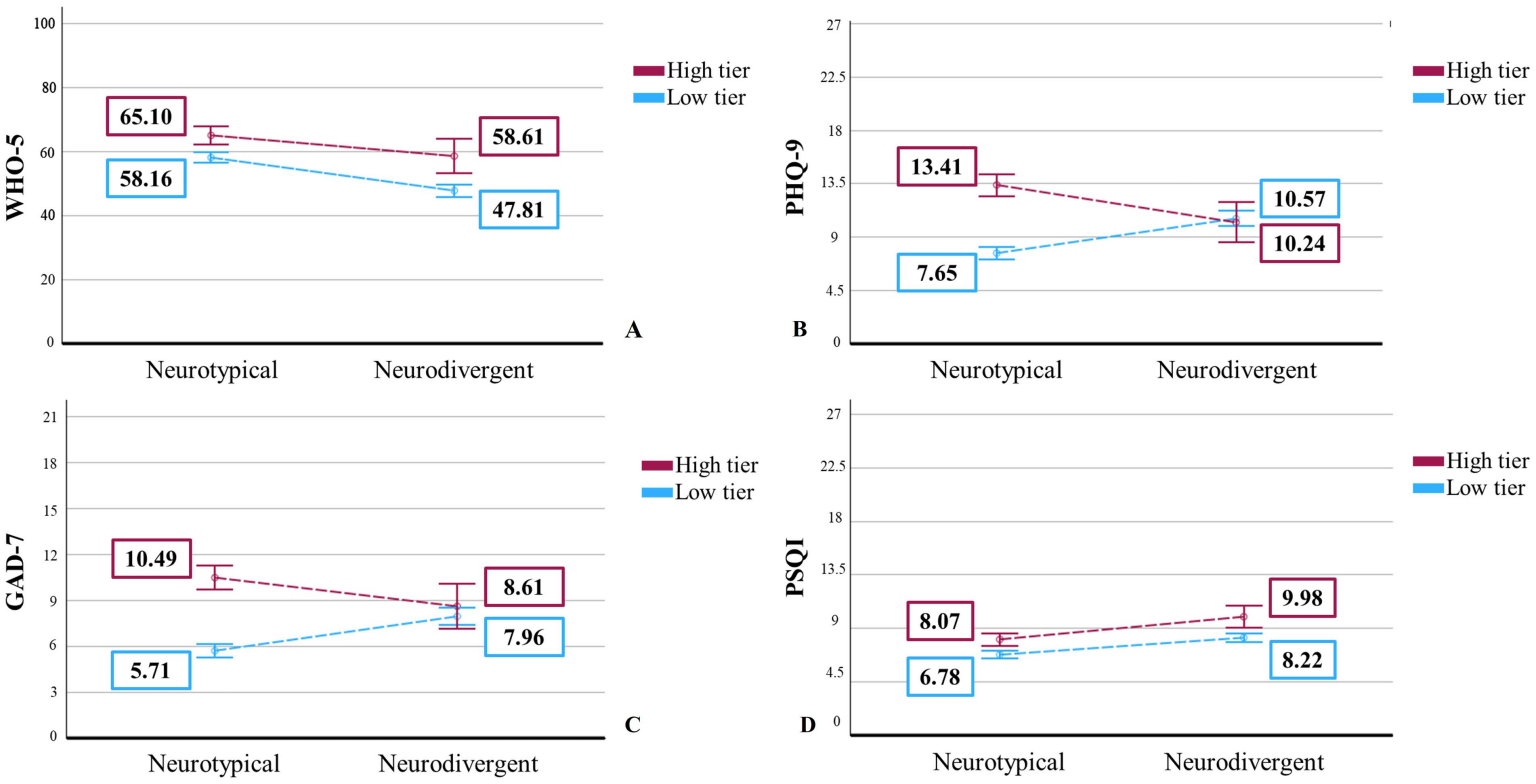
Estimated marginal means of mental health outcomes by neurotype and competitive tier. Estimated marginal means are shown for **(A)** well-being measured with the WHO-5, **(B)** depressive symptoms measured with the PHQ-9, **(C)** anxiety symptoms measured with the GAD-7, and **(D)** sleep disturbance measured with the PSQI. Error bars represent 95% confidence intervals around the mean estimates. Low tier includes recreational, foundational, and pre-elite players, high tier includes elite and mastery players. Higher WHO-5 scores indicate greater well-being, whereas PHQ-9, GAD-7, and PSQI scores indicate greater symptom severity or sleep disturbance.

To examine group differences in well-being, depression, anxiety, and sleep disturbance, we conducted four 2 × 2 factorial ANOVAs with neurotype (neurodivergent–neurotypical) and competitive tier (“low”–“high”) as between subject factors. Levene’s tests for all outcomes were significant (WHO-5: *F*[3, 1,071] = 3.22, *p* = 0.02; PHQ-9: *F*[3, 1,071] = 2.70, *p* = 0.04; GAD-7: *F*[3, 1,071] = 4.35, *p* = 0.005; PSQI: *F*[3, 1,071] = 5.09, *p* = 0.002), but due to the large sample size, the ANOVAs were retained (Blanca et al., 2017). Figure 1 presents the estimated marginal means for each outcome by neurotype and tier (error bars indicate the 95% CIs around the mean estimates).

For well-being (WHO-5), the 2 × 2 ANOVA (Figure 1, panel A) showed a main effect of neurotype (*F*[1, 1,071] = 25.15, *p* < 0.001, partial *η*^2^ = 0.023), a main effect of competitive tier (*F*[1, 1,071] = 27.94, *p* < 0.001, partial *η*^2^ = 0.025), and no interaction effect (*F*[1, 1,071] = 1.32, *p* = 0.25, partial *η*^2^ = 0.001). Within the “high” competitive tier, neurodivergent players reported significantly lower well-being than their neurotypical counterparts (mean difference [MD] = −6.49, SE = 3.08, p = 0.04, 95% CI [−12.55, −0.43]). Similarly, within the “low” competitive tier, neurodivergent players reported significantly lower well-being than their neurotypical counterparts (MD = −10.35, SE = 1.32, *p* < 0.001, 95% CI [−12.94, −7.76]). Comparing neurodivergent players across competitive tiers, “high-tier” neurodivergent players reported significantly higher well-being than their “low-tier” neurodivergent counterparts (MD = 10.81, SE = 2.89, *p* < 0.001, 95% CI [5.12, 16.49]).

For depression (PHQ-9), the 2 × 2 ANOVA (Figure 1, panel B) showed no main effect of neurotype (*F*[1, 1,071] = 0.05, *p* = 0.82, partial *η*^2^ = 0.00), a main effect of competitive tier (*F*[1, 1,071] = 25.55, *p* < 0.001, partial *η*^2^ = 0.023), and an interaction effect (*F*[1, 1,071] = 31.96, *p* < 0.001, partial *η*^2^ = 0.029). Within the “high” competitive tier, neurodivergent players reported significantly lower PHQ-9 scores compared to their “high-tier” neurotypical counterparts (MD = −3.16, SE = 0.99, *p* = 0.001, 95% CI [−5.1, −1.22]). Conversely, within the “low tier,” neurodivergent players reported significantly higher PHQ-9 scores than their “low-tier” neurotypical counterparts (MD = 2.91, SE = 0.42, *p* < 0.001, 95% CI [2.09, 3.74]). Comparing neurodivergent players across competitive tiers, PHQ-9 scores did not significantly differ between “high” and “low” competitive tier neurodivergent individuals (MD = −0.32, SE = 0.93, *p* = 0.73, 95% CI [−2.14, 1.49]).

For anxiety (GAD-7), the 2×2 ANOVA (Figure 1, panel C) showed no main effect of neurotype (*F*[1, 1,071] = 0.16, *p* = 0.69, partial *η*^2^ = 0.00), a main effect of competitive tier (*F*[1, 1,071] = 34.83, *p* < 0.001, partial *η*^2^ = 0.031), and an interaction effect (*F*[1, 1,071] = 20.06, *p* < 0.001, partial *η*^2^ = 0.018). Within the “high tier,” neurodivergent players reported significantly lower GAD-7 scores compared to their neurotypical counterparts (MD = −1.88, SE = 0.85, *p* = 0.03, 95% CI [−3.54, −0.22]). Conversely, within the “low tier,” neurodivergent individuals reported significantly higher GAD-7 scores compared to their neurotypical counterparts (MD = 2.25, SE = 0.36, *p* < 0.001, 95% CI [1.54, 2.96]). Comparing neurodivergent players across competitive tiers, GAD-7 scores did not significantly differ between “high” and “low” tier neurodivergent players (MD = 0.66, SE = 0.79, *p* = 0.41, 95% CI [−0.91, 2.21]).

Finally, for sleep disturbance (PSQI), the 2 × 2 ANOVA (Figure 1, panel D) showed a main effect of neurotype (*F*[1, 1,071] = 30.62, *p* < 0.001, partial *η*^2^ = 0.028), a main effect of competitive tier (*F*[1, 1,071] = 25.59, *p* < 0.001, partial *η*^2^ = 0.023), and no interaction effect (*F*[1, 1,071] = 0.62, *p* = 0.43, partial *η*^2^ = 0.001). Within the “high tier,” neurodivergent players reported significantly higher PSQI scores compared to their neurotypical counterparts (MD = 1.91, SE = 0.56, *p* < 0.001, 95% CI [0.82, 2.99]). Similarly, within the “low tier,” neurodivergent players reported significantly higher PSQI scores compared to their neurotypical counterparts (MD = 1.43, SE = 0.24, *p* < 0.001, 95% CI [0.97, 1.89]). Comparing neurodivergent players across tiers, “high-tier” neurodivergent players reported significantly higher PSQI scores than their “low-tier” counterparts (MD = 1.76, SE = 0.52, *p* < 0.001, 95% CI [0.74, 2.79]).

## 4 Discussion

The aim of this study was to examine how neurotype and self-reported competitive tier were jointly associated with mental health outcomes in esports. Approximately 37% of esports players self-identified as neurodivergent, with greater representation at the lower competitive levels, and ADHD (18.3%) and autism (9.6%) were reported most frequently. Across the sample, neurodivergent players reported lower well-being and greater sleep disturbance, while depressive and anxiety symptoms did not differ between neurotypes. Moreover, consistent with the original article by Kegelaers et al. (2025), high-tier players generally reported more depressive symptoms, more anxiety symptoms, and greater sleep disturbance, while also reporting higher well-being compared to low-tier players. While these differences describe average trends across the whole sample, they do not capture the more nuanced neurotype differences.

Across competitive tiers, low-tier neurodivergent players showed the most unfavourable mental health profile, reporting lower well-being, higher depressive and anxiety symptoms, and greater sleep disturbance compared to their low-tier neurotypical counterparts. One potential explanation may be that this finding reflects general population disparities, as neurodivergent individuals often report elevated levels of mental ill-health (Hollocks et al., 2019; Meinzer et al., 2016). At the same time, it is plausible that the lower-tier esports context may amplify existing mental health issues in lower-tier neurodivergent players. Compared with more formalised competitive settings, low-tier environments may be less predictable and less structured, with more ambiguous role expectations and less consistent access to support or accommodations (Leis et al., 2025). In addition, some neurodivergent individuals may be drawn to gaming/esports for coping- or self-regulation-oriented reasons. For some, gaming may provide predictability, stress relief, or a manageable social context rather than constituting a risk in itself (Finke et al., 2018; Mazurek et al., 2015). However, when gaming/esports serves primarily avoidant or escapist functions, it has been associated with greater distress (Bányai et al., 2019, 2021). Taken together, one possible explanation for the observed low-tier pattern is that it may reflect a combination of pre-existing mental health disparities and contextual features of low-tier esports that can create a poorer person-environment fit for some neurodivergent individuals.

A notable finding was that the neurotype pattern for depressive and anxiety symptoms differed across competitive tiers. As mentioned, the low-tier neurodivergent players reported higher depressive and anxiety symptoms than low-tier neurotypical players. At the higher competitive tier, however, neurodivergent players reported lower depressive and anxiety symptoms than their high-tier neurotypical counterparts. In contrast, the neurotype pattern for lower well-being and elevated sleep disturbance experienced by neurodivergent players was more consistent across tiers. Taken together, the findings suggest a more nuanced profile (i.e., lower well-being and higher sleep disturbance alongside lower levels of anxiety and depressive symptoms) among high-tier neurodivergent players relative to their high-tier neurotypical counterparts.

One possible explanation for the comparatively more favourable depressive and anxiety profile observed among high-tier neurodivergent players is that it reflects differences in the environments in which they compete. High-tier environments may provide greater structure, clearer roles, and more consistent access to resources and embedded support systems (e.g., coaching, performance support, stable team routines), which may be particularly beneficial and accommodating for neurodivergent individuals (Hoare et al., 2023). These features may help reduce ambiguity and support day-to-day life, which could in turn relate to better well-being and lower distress as a result of environment mismatches for some neurodivergent players.

An alternative, yet not mutually exclusive, explanation may lie in selection or attrition processes. If neurodivergence is accompanied by an elevated mental health burden, this compounding burden may make it harder to progress into, or sustain, participation at high competitive levels. As players move into elite settings, demands related to communication, team coordination, travel, and media exposure often increase (Leis et al., 2024; Pedraza-Ramirez et al., 2024). For some neurodivergent individuals, these intensifying demands may become too challenging to navigate if environments fail to accommodate diverse communication styles, sensory preferences, or executive profiles (Wood et al., 2025). In other words, there might be a survivorship bias, whereby co-occurring neurodivergence and mental ill-health symptoms may reduce the likelihood of progression to, or retention in, higher-tier competition for some neurodivergent players. This interpretation aligns with evidence from traditional sports contexts suggesting that neurodivergent athletes may face organisational and interpersonal barriers, misunderstanding, and limited support, all of which may contribute to increased strain, attrition, or reduced progression (Hoare et al., 2023; Ruffino et al., 2025; Wood et al., 2025). This might also explain why neurodivergent players were comparatively less represented at the high competitive tier, compared to the low tier. Despite these differences in representation, it should still be noted that the prevalence of neurodivergence across both groups appears elevated relative to general population estimates (Francés et al., 2022). This suggests that the esports performance context can be a well-matched environment for many neurodivergent individuals to thrive in.

### 4.1 Practical implications

Our findings carry several implications for esports teams, organisations, and support staff. Given that more than one in three players in our sample identified as neurodivergent, and that the low-tier neurodivergent subgroup reported a less favourable mental health profile, neurodivergence should be treated as a routine consideration in player welfare and performance systems. Teams and organisations can adopt neurodiversity-informed practices such as clear and concrete communication, predictable scheduling, minimising unnecessary last-minute changes, and actively promoting psychologically safe environments (Hoare et al., 2023). A neurodiversity-informed approach should also be strengths-based, matching roles and routines to individual profiles while reducing avoidable friction. In parallel, coaches, performance staff, or psychologists working with esports players should receive training in neurodiversity-informed practice. Neurodivergence can be associated with an elevated mental health risk for some players and may require adjustment of assessment and intervention approaches (e.g., different communication formats, greater structure, and context-specific accommodations). Embracing these inclusive practices may support the well-being of neurodivergent players, reduce avoidable stress, and enhance performance outcomes.

### 4.2 Strengths and limitations

This study benefitted from a large, international sample spanning the full competitive spectrum and a wide array of game titles. Our study aligned with a dual-continuum framework considering both positive well-being and psychological distress (Keyes, 2002), and used well-validated measures of well-being, depression, anxiety, and sleep disturbance. Moreover, to our knowledge, this is the first paper studying neurodivergence and mental health in esports. However, important limitations must also be acknowledged. Neurotype was assessed using a single self-report item, yielding a heterogeneous neurodivergence group that included potentially diagnosed, self-identified, and broadly defined neurotypes. Relatedly, we collapsed distinct profiles (e.g., ADHD, autism, dyslexia) into a single category, limiting condition specific inference. Competitive level was self-reported, and definitional challenges around “elite” esports status has been noted (Poulus et al., 2024). Relatedly, although the sample included players from a wide range of game titles, genre-specific analyses remained limited to descriptive reporting. Game genres differ in cognitive, social, and tactical demands, and these differences may be relevant to mental health outcomes. Future research may purposefully sample to examine whether neurotype-related mental health profiles differ within and across specific esports titles and genres. The cross-sectional design precludes causal inference and there may be risk of self-selection bias as individuals who already have an interest in mental health might be more likely to complete the survey. Finally, the sample consisted predominantly of men. Caution is, therefore, warranted when generalising findings to women, non-binary players, or other underrepresented genders in esports.

### 4.3 Future directions

Future research would benefit from more nuanced and inclusive approaches to capture neurodivergence. Although we provided a descriptive breakdown of neurotype co-occurrence, the present study was not powered to compare specific neurotype profiles or combinations. Future research should move beyond broad neurodivergent-neurotypical comparisons and examine specific and co-occurring profiles. Longitudinal designs are needed to clarify temporal relationships between neurotype, mental health, and progression/attrition across competitive levels, while qualitative and mixed-methods studies should examine the lived experiences, environmental barriers, and supports of neurodivergent players. Finally, research examining inclusive and neuro-affirmative coaching and the implementation of organisational practices may help identify ways to foster sustainable participation, reduce unnecessary stress, and support both well-being and performance in esports.

### 4.4 Conclusion

Neurodivergence appears to be a salient dimension of esports participation. A substantial proportion of players in the current sample self-identified as neurodivergent, and group-level differences were observed in mental health outcomes and representation at the highest competitive levels. Competitive tier-based analyses further indicated that the mental health profile associated with neurodivergence was not uniform across the competitive pathway. These findings suggest that aspects of competitive esports environments may differentially shape experiences and progression for neurodivergent players. Continued research and the development of inclusive, neurodiversity-affirming policies are, therefore, needed to better support well-being, remove unnecessary barriers, and ensure esports is an environment where neurodivergent players can thrive.

## Data Availability

The datasets presented in this article are not readily available because data will be stored in a public repository upon the completion of the project. Data will be available upon reasonable request. Requests to access the datasets should be directed to Lucas Van Ruysevelt, lucas.van.ruysevelt@vub.be.
