# Supplementary material for: Neurodiversity and mental health in esports
Source: Front Psychol. 2026 Jun 1;17:1843950. doi: 10.3389/fpsyg.2026.1843950 (PMC13265385; doi:10.3389/fpsyg.2026.1843950)
Supplement: Supplementary file 1 [file Table_1.DOCX]

Supplementary Material

# Supplementary Tables

**Table S1.**

Distribution of game titles and game genres, and their corresponding mean mental health outcomes, low tier-high tier distribution, and neurotype distribution

| **Genre** | **Game title** | **Count (%)** | **WHO-5 (SD)** | **PHQ-9 (SD)** | **GAD-7 (SD)** | **PSQI (SD)** | **Low Tier *n* (High Tier *n*)** | **Neurodivergent *n* (%)** |
| --- | --- | --- | --- | --- | --- | --- | --- | --- |
| **Shooter** | --- | **312 (29.02%)** | **57.44 (19.8)** | **10.66 (6.39)** | **8.34 (5.61)** | **8.11 (3.49)** | **223 (89)** | **132 (42.31%)** |
|  | CS:GO | 81 (7.5%) | 54.52 (21.16) | 9.99 (6.28) | 7.93 (5.44) | 7.89 (3.30) | 70 (11) | 32 (39.51%) |
|  | Rainbow Six | 55 (5.1%) | 60.96 (15.36) | 11.78 (6.93) | 9.60 (5.50) | 8.42 (3.87) | 28 (27) | 17 (30.91%) |
|  | Overwatch | 59 (5.5%) | 53.64 (20.04) | 9.75 (5.46) | 7.00 (5.59) | 7.78 (3.15) | 45 (14) | 34 (57.63%) |
|  | Valorant | 52 (4.8%) | 58.84 (18.28) | 10.77 (6.33) | 8.04 (5.78) | 7.79 (3.45) | 37 (15) | 30 (57.69%) |
|  | Fortnite | 47 (4.4%) | 62.56 (22.16) | 11.98 (6.67) | 9.81 (5.35) | 8.34 (3.34) | 29 (18) | 11 (23.40%) |
|  | Call of Duty | 18 (1.7%) | 54.88 (18.36) | 9.56 (5.98) | 7.78 (6.18) | 9.61 (4.49) | 14 (4) | 8 (44.44%) |
| **MOBA** | --- | **412 (38.3%)** | **53.84 (20.08)** | **9.27 (6.30)** | **7.04 (5.14)** | **7.25 (3.32)** | **355 (57)** | **139 (33.74%)** |
|  | League of Legends | 331 (30.8%) | 53.56 (19.80) | 9.41 (6.22) | 7.15 (5.13) | 7.17 (3.20) | 287 (44) | 115 (34.74%) |
|  | DOTA2 | 81 (7.5%) | 55.00 (21.24) | 8.72 (6.64) | 6.58 (5.15) | 7.59 (3.77) | 68 (13) | 24 (29.63%) |
| **Sports** | --- | **96 (8.93%)** | **62.72 (19.68)** | **12.53 (7.19)** | **9.94 (5.86)** | **8.39 (4.16)** | **45 (51)** | **22 (22.92%)** |
|  | Rocket League | 38 (3.5%) | 63.56 (18.24) | 13.82 (7.20) | 10.24 (5.76) | 9.39 (4.16) | 14 (24) | 6 (15.79%) |
|  | EA Sports FC | 39 (3.6%) | 61.64 (20.60) | 12.21 (7.50) | 9.67 (5.97) | 8.05 (3.93) | 22 (17) | 7 (17.95%) |
|  | NBA2K | 10 (0.9%) | 66.40 (13.88) | 13.70 (7.02) | 12.20 (6.83) | 7.90 (5.61) | 4 (6) | 4 (40.00%) |
|  | Sim racing | 9 (.08%) | 55.56 (28.12) | 7.22 (3.31) | 7.33 (4.27) | 6.11 (2.15) | 5 (4) | 5 (55.55%) |
| **Other** | --- | **255 (23.7%)** | **54.60 (19.36)** | **7.82 (5.77)** | **5.55 (5.10)** | **7.24 (3.50)** | **236 (19)** | **109 (42.75%)** |

**Table S2.**

Prevalence of neurodiversity across performance levels and 2x2 pairwise comparisons between adjacent performance levels

| **Comparison** | **% Neurodivergence in group 1** | **% Neurodivergence in group 2** | **χ²** | **Odds ratio** | **95% CI** | **p** |
| --- | --- | --- | --- | --- | --- | --- |
| Recreational- Foundational | 42% | 39.9% | .27 | .92 | [.67, 1.26] | .61 |
| Foundational – Pre-elite | 39.9% | 41.3% | .10 | 1.06 | [.75, 1.50] | .75 |
| Pre-elite - Elite | 41.3% | 23.8% | 12.15 | .44 | [.28, .70] | < .001 |
| Elite - Mastery | 23.8% | 20.3% | .33 | .82 | [.41, 1.64] | .57 |

**Table S3.**

Descriptive prevalences of single specified, multiple specified, and non-specified/uncodable neurodivergent profiles and their corresponding mean mental health outcomes

| **Profile type** | **Neurotype profile** | **Count (% of ND group)** | **WHO-5 (SD)** | **PHQ-9 (SD)** | **GAD-7 (SD)** | **PSQI (SD)** |
| --- | --- | --- | --- | --- | --- | --- |
| Single specified  profile | --- | **218 (54.2%)** | **48.84 (18.64)** | **9.98  (5.85)** | **7.61 (5.24)** | **7.82 (3.22)** |
|  | ADHD only | 126 (31.3%) | 47.56 (18.12) | 10.29 (5.79) | 7.87 (4.96) | 8.00 (3.21) |
|  | Autism only | 49 (12.2%) | 51.04 (19.16) | 9.39  (5.64) | 7.39 (5.50) | 7.59 (3.11) |
|  | Dyslexia/learning-related only | 27 (6.7%) | 51.24 (20.24) | 9.62  (6.67) | 6.77 (6.17) | 7.35 (3.51) |
|  | Other single only | 16 (4.0%) | --- | --- | --- | --- |
| Multiple specified profile | --- | **83 (20.7%)** | **42.80 (19.92)** | **11.55 (6.54)** | **8.74 (5.81)** | **8.58 (3.38)** |
|  | ADHD + Autism | 44 (11.0%) | 43.64 (18.76) | 11.55 (5.89) | 8.09 (4.92) | 8.68 (3.38) |
|  | ADHD + Dyslexia/learning-related | 14 (3.5%) | 39.52 (22.88) | 12.18 (8.30) | 10.29 (7.44) | 8.18 (3.71) |
|  | Other multiple | 25 (6.2%) | 44.00 (20.52) | 10.87 (6.53) | 8.87 (6.22) | 8.73 (3.15) |
| Other (non-specified or uncodable) | --- | 101 (25.1%) | 54.44 (22.32) | 11.14 (6.92) | 8.60 (5.81) | 9.75 (4.08) |
